# IPFSCNN: A Time–Frequency Fusion CNN for Wideband Spectrum Sensing

**DOI:** 10.3390/s25237134

**Published:** 2025-11-22

**Authors:** Soon-Young Kwon, Do-Hyun Park, Hyoung-Nam Kim

**Affiliations:** School of Electrical and Electronics Engineering, Pusan National University, Busan 46241, Republic of Korea; ysk1680@pusan.ac.kr (S.-Y.K.);

**Keywords:** wideband spectrum sensing, IQ/FFT fusion, cognitive radio

## Abstract

Wideband spectrum sensing is a crucial technology for the efficient utilization of limited frequency resources in cognitive radio. While deep learning models have yielded promising results, they typically rely on either time-domain (I/Q) or frequency-domain (FFT) data alone, which can limit their performance. This study proposes IPFSCNN (IQ-Parallel FFT-Serial CNN), a novel asymmetric hybrid architecture that synergistically fuses both data representations. The key idea of its design is an asymmetric architecture that employs two specialized streams: a parallelized branch to efficiently capture temporal features from I/Q data, and a deep serial branch to extract spectral patterns from FFT data. These complementary features are fused to perform a multi-label classification task. Experiments on an LTE-M dataset demonstrate that the proposed IPFSCNN achieves a higher detection performance than state-of-the-art models, including DeepSense and ParallelCNN, particularly in low signal-to-noise ratio conditions. Furthermore, IPFSCNN achieves this superior accuracy while maintaining high computational efficiency, requiring 15% fewer parameters and only one-third of the multiply-accumulate (MAC) operations compared to the DeepSense model. Crucially, a comprehensive ablation study validates this asymmetric design, proving that the proposed ‘IQ-Parallel FFT-Serial’ combination is demonstrably superior to other hybrid configurations.

## 1. Introduction

The rapid growth of 5th generation (5G) technology and the Internet of Things (IoT) presents a significant challenge to the limited availability of radio spectrum [1,2,3]. Cognitive radio (CR) has emerged as a key technology to solve this problem by enabling dynamic and opportunistic access to underutilized frequency bands [4,5,6]. The successful implementation of CR is essential for efficient spectrum use and relies heavily on spectrum sensing—the ability to quickly and accurately detect unoccupied frequencies, or “spectrum holes.” However, performing this task in real time is a considerable technical challenge, compounded by high computational requirements and low signal-to-noise ratio (SNR) conditions [7,8,9].

Spectrum sensing techniques are generally categorized into narrowband and wideband approaches. Narrowband approaches detect the presence of a signal in a single channel at a time. In contrast, wideband spectrum sensing, which simultaneously monitors a wide spectrum to locate multiple holes, is considered essential for modern CR applications [10]. Conventional approaches to wideband spectrum sensing have relied on methods such as energy detection, matched filtering, and compressive sensing [11,12,13,14]. While energy detection is simple to implement, its performance degrades significantly in low-SNR scenarios [11]. Other techniques like matched filtering can perform well but require prior knowledge of the primary user’s signal, limiting their applicability in diverse and unknown signal environments [12]. Compressive sensing techniques aim to reduce the required sampling rate but often introduce high latency and complexity due to the computationally intensive signal reconstruction process, which can also degrade sensing performance [13,14]. These limitations have motivated the exploration of data-driven approaches that can build robust and adaptive solutions without relying on predefined signal models [15,16,17].

In recent years, deep learning methods, particularly convolutional neural networks (CNNs), have been widely studied for spectrum sensing applications [18,19]. Foundational work by Uvaydov et al. introduced DeepSense [18], a serial CNN model that demonstrated the feasibility of real-time sensing by processing raw I/Q samples directly. This model was designed for real-time performance with a lightweight architecture, extracting features directly from the I/Q waveform to avoid complex preprocessing. Building on this, Mei and Wang proposed ParallelCNN [19], which partitions the input I/Q data for parallel processing. This architectural design aims to overcome the sequential processing bottlenecks of a single deep network and effectively utilize the parallel processing power of modern hardware, leading to reduced latency.

Despite these advances, a notable limitation of these models is their exclusive reliance on time-domain I/Q data. While I/Q data contains the complete signal information, the neural network must undertake the complex task of learning to extract frequency-related features from the time-domain waveform. In contrast, frequency-domain data obtained from a fast Fourier transform (FFT) offers a direct representation of the energy distribution across the spectrum, which is highly effective for determining occupancy. While the use of such FFT data has been well-explored for narrowband spectrum sensing [20,21], deep learning architectures for wideband spectrum sensing that simultaneously fuse both I/Q and FFT data remain relatively under-investigated.

To address this limitation in wideband spectrum sensing, we propose a novel dual-stream architecture, the IPFSCNN (IQ-Parallel FFT-Serial CNN), which is designed for the synergistic integration of both I/Q and FFT data. The proposed model features two distinct extraction branches:

- IQ-Parallel Branch: To effectively capture complex temporal features from the raw I/Q signal with low latency, this branch employs a parallel processing strategy. The time-domain signal is partitioned into two segments for concurrent processing by two CNN blocks. This architectural choice is highly effective for extracting dynamic characteristics, such as phase and amplitude variations.
- FFT-Serial Branch: This branch analyzes the FFT data using a single, deeper serial CNN. As the spectral shape, energy distribution, and band characteristics all differ depending on the occupancy state of each sub-band, the CNN is trained to learn this direct correspondence.

The feature vectors extracted from both branches are ultimately combined into a fully connected layer, which makes a final, comprehensive judgment based on information from both the time and frequency domains.

The primary contributions of this work are as follows:

1. We analyze and identify the performance discrepancy of deep learning models for spectrum sensing, which arises from the specific combination of input data representation (I/Q vs. FFT) and model architecture (serial vs. parallel).
2. We propose a novel hybrid dual-stream architecture, IPFSCNN, that synergistically learns from both time-domain and frequency-domain information in a complementary manner.
3. We experimentally demonstrate that the proposed model can achieve higher accuracy than state-of-the-art models that rely on a single data representation. Furthermore, we validate the effectiveness of our proposed architecture through a comprehensive ablation study, proving that our asymmetric hybrid (IQ-Parallel FFT-Serial) design is superior to other combinations.

The remainder of this paper is organized as follows. Section 2 details the architecture of the proposed IPFSCNN model. Section 3 describes the experimental setup, dataset, and performance metrics. Section 4 presents our experimental results, including a comparative performance analysis and an ablation study of the proposed model. Finally, Section 5 concludes the paper and discusses future work.

## 2. Proposed Model

**Figure 1:**
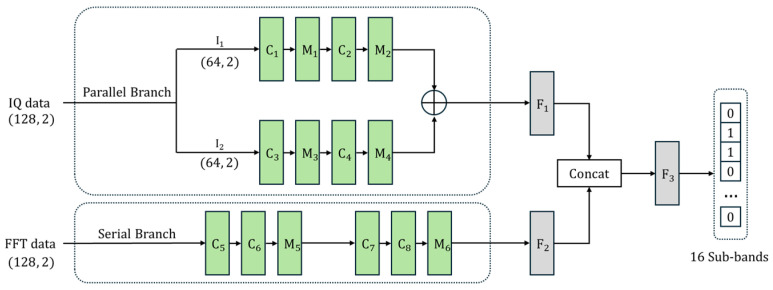
IPFSCNN architecture.

To effectively address the challenges of wideband spectrum sensing, we propose a novel deep learning architecture, the IPFSCNN (IQ-Parallel FFT-Serial CNN). This model is designed to synergistically leverage the complementary strengths of both time-domain and frequency-domain data representations. The core of the IPFSCNN is its dual-stream design, which processes raw I/Q data and FFT data through specialized branches before fusing their extracted features for a final classification. The overall architecture is depicted in Figure 1, with detailed layer configurations specified in Table 1.

### 2.1. IQ-Parallel Branch

The Parallel Branch is specifically designed to capture changing and time-based features from the raw I/Q waveform, while also keeping the processing time low. As shown in Figure 1, the input I/Q data (128, 2) is first split into two equal segments, I1 and I2, each with a shape of (64, 2). These segments are then processed at the same time by two identical and relatively simple CNN pathways. Each pathway contains two main blocks:

- The first 1D convolutional layer (C1/C3) uses 8 filters with a kernel size of 3 and a stride of 1, along with a LeakyReLU activation function with a negative slope of 0.2. As the CNN kernels convolve over the I/Q data sequence, they learn to identify diverse temporal features such as amplitude variations, phase changes, and zero-crossing patterns which differ depending on the occupancy state of the various sub-bands.
- The next max-pooling layer (M1/M3) has a kernel size and stride of 2, which halves the length of the sequence to (31, 8).
- The second 1D convolutional layer (C2/C4) uses 32 filters with a kernel size of 5 and a stride of 2. This step further reduces the data size and allows the model to learn more complex time-based patterns.
- A final max-pooling layer (M2/M4) completes the process, resulting in an output shape of (7, 32) for each pathway.

The resulting feature maps from these two parallel pathways are then combined using an element-wise addition operation. This is a fast and efficient step that merges the features learned from two back-to-back time windows into a single time-domain feature representation.

### 2.2. FFT-Serial Branch

The Serial Branch is designed to analyze the overall and large-scale patterns found in the frequency spectrum of the entire signal. The input for this branch is the FFT data from all 128 samples, which provides a clear representation of how the signal energy is spread across the frequency band.

This branch uses a deeper, serial CNN architecture that is well-suited for spectral data, as shown in Figure 1 and Table 1.

- The first block of 1D convolutional layers (C5, C6) uses 8 filters with a kernel size of 5 to begin identifying basic spectral shapes.
- A max-pooling layer (M5) then downsamples the feature map.
- The next block of 1D convolutional layers (C7, C8) uses 16 filters with a larger kernel size of 11. This larger size is important for capturing wider patterns, such as the shape of an entire channel.
- A final max-pooling layer (M6) reduces the dimension to a final feature map of shape (20, 16).

This deeper, serial design is built to extract key frequency-domain patterns like channel energy, bandwidth, and spectral shape from the FFT data.

### 2.3. Feature Fusion and Classification Stage

The final stage of IPFSCNN is designed to integrate the complementary features of the two specialized branches to make a final decision. As shown in Figure 1, features from the parallel and serial branches are first independently refined in fully connected (dense) layers and then combined.

- Features from the parallel branch are flattened into a 224-dimensional vector and fed into the first dense layer (F1). This layer converts temporal information into a compact 64-dimensional feature vector. Similarly, features from the serial branch are flattened and processed in the second dense layer (F2) to produce a 32-dimensional feature vector representing key spectral information. These initial dense layers (F1 and F2) serve to summarize features by generating representations for each domain before the main fusion step.
- Next, the two resulting vectors are combined using a concatenation operation, as shown in Figure 1. The 64-dimensional time-domain vector and the 32-dimensional frequency-domain vector are combined to form a single 96-dimensional vector. This approach preserves all information learned from both streams, allowing the final layer to learn complex connections between time-based and frequency-based patterns.
- The resulting 96-dimensional vector is processed by the final classifier (F3), designed for multi-label classification tasks in wideband spectrum sensing. The output layer consists of 16 neurons, each corresponding to one of 16 sub-bands within the wideband spectrum. This layer uses a sigmoid activation function to generate a probability score between 0 and 1 for each sub-band. This score represents the probability that the sub-band is occupied. To make a final binary decision, these probability scores are compared against a predefined threshold. If the sub-band’s score exceeds the threshold, it is classified as occupied (1); otherwise, it is classified as unoccupied (0). This final thresholding step produces a binary output vector, as shown in the last part of Figure 1.

## 3. Experimental Setup

This section details the experimental environment, including the dataset and the specific metrics used to evaluate and compare the performance of the proposed IPFSCNN model.

### 3.1. LTE-M Dataset

**Figure 2:**
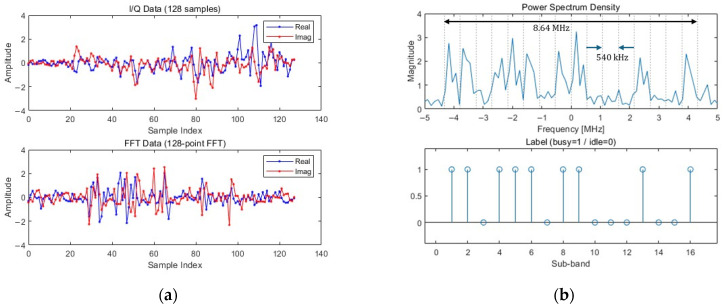
LTE-M dataset example at SNR 10 dB: (**a**) I/Q data and FFT data. (**b**) Frequency spectrum and label.

To ensure a precise evaluation, all experiments in this study were conducted using LTE-M dataset generated with MATLAB R2024a LTE Toolbox. This dataset is the same as that used in the baseline studies, DeepSense and ParallelCNN, allowing for a fair and direct comparison of the performance improvement of our proposed model against the existing models. The dataset was designed to simulate the characteristics of a realistic wireless environment, consisting of LTE-M uplink signals on the physical uplink shared channel (PUSCH). The signal has a total bandwidth of 8.64 MHz and was generated using a sampling frequency of 15.36 MHz. This entire band of 8.64 MHz is divided into 16 non-overlapping sub-bands, each with a bandwidth of 540 kHz. Since each of the 16 sub-bands can be either occupied or unoccupied by primary users (PUs), this results in $2^{16}$ unique occupancy scenarios. The generated dataset consists of I/Q data, FFT data, and corresponding labels. Figure 2a shows an example of the I/Q and FFT data, while Figure 2b illustrates a corresponding label. Specifically, each signal in the dataset consists of a sequence of 128 I/Q data points. This sequence serves as the direct input for the IQ-Parallel branch, while the input for the FFT-Serial branch is generated by applying a 128-point FFT to the same sequence. The label is a 16-element binary vector $Y=[y_{1},\cdots ,y_{i},\cdots ,y_{16}]$, where $y_{i}=1$ if the i-th sub-band is occupied and $y_{i}=0$ i-th sub-band is unoccupied. To emulate non-line-of-sight (NLOS) channel conditions, all transmission signals were passed through a Rayleigh fading channel and subjected to additive white Gaussian noise (AWGN). A comprehensive collection of 65,536 transmission samples per SNR was generated across a wide range of SNR levels, from −20 dB to 10 dB, to thoroughly assess the models’ robustness against noise. To maintain consistency and stability during the training process, the average power of all input signals was normalized to 1.

### 3.2. Model Training and Evaluation Metrics

The entire dataset was partitioned into training, validation, and test sets using an 80%, 10%, and 10% split, respectively. All models were trained using the Adam optimizer. As wideband spectrum sensing is a multi-label classification problem, the binary cross-entropy (BCE) was employed as the loss function. To prevent overfitting and select the best-performing model, we utilized an early stopping criterion.

To provide an assessment of model performance, we employed several standard evaluation metrics. The performance of the models is evaluated based on true positives (TP), false positives (FP), and false negatives (FN), where TP is the number of correctly identified occupied channels, FP is the number of unoccupied channels incorrectly identified as occupied, and FN is the number of occupied channels missed by the model.

Since the spectrum sensing task is framed as a multi-label classification problem, we use the micro-averaged Precision, Recall, and F1-Score. Precision measures the accuracy of the positive predictions, while Recall measures the model’s ability to find all occupied channels. They are defined as follows:

(1) $$Precision=\frac{True\;Positives}{True\;Positives+False\;Positives}$$

(2) $$Recall=\frac{True\;Positives}{True\;Positives+False\;Negatives}$$

The F1-Score, which is the harmonic mean of Precision and Recall, serves as the primary metric for representing overall model accuracy. It provides a balanced measure of the two and is calculated as

(3) $$F1=2\times \frac{Precision\times Recall}{Precision+Recall}$$

In addition to overall accuracy, we evaluated the practical detection capability of the models using the receiver operating characteristic (ROC) curve. The ROC curve provides a crucial visualization of the trade-off between the probability of detection ($P_{d}$) and the probability of false alarm ($P_{fa}$). By comparing the $P_{d}$ achieved at a specific $P_{fa}$, we can assess a model’s robustness and its reliability as a practical detection system in noisy conditions.

## 4. Simulation Results

In this section, we present and analyze the simulation results to quantitatively evaluate the performance of the proposed IPFSCNN model and to validate its architectural design. The experiments include a comparative performance analysis against state-of-the-art models, an assessment of robustness in low-SNR environments, and an ablation study to evaluate the contribution of the model’s key components.

### 4.1. Comparative Performance Analysis

**Figure 3:**
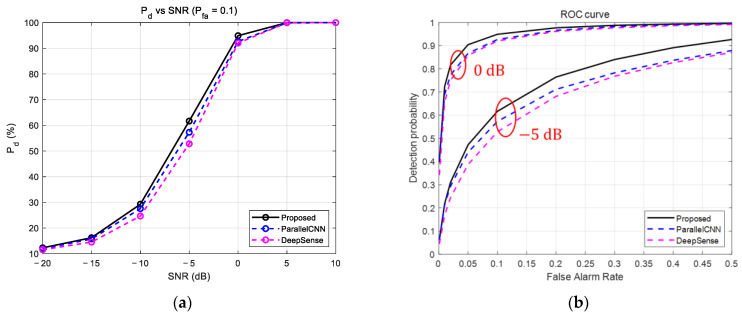
Performance comparison of the proposed model with baseline models: (**a**) Detection probability versus SNR at a false alarm rate of 0.1. (**b**) ROC curve comparison at an SNR of 0, −5 dB.

As shown in Figure 3a, a performance gap between the models emerged in the low-SNR region (especially from −15 dB to 0 dB), where the proposed model demonstrated a 3% to 9% higher detection performance than the other two models. Notably, at an SNR of −5 dB, the proposed model’s performance was approximately 5% superior to the ParallelCNN model and 9% superior to the DeepSense model.

Figure 3b provides a more detailed view of the models’ detection performance through ROC curves at SNR conditions of 0 dB and −5 dB. It is evident that the proposed model’s curve is positioned closest to the top-left, indicating the best performance among the three. This means that for the same $P_{fa}$, the proposed model achieves a higher $P_{d}$. For example, at SNR 0 dB, when $P_{fa}$ is 0.1, the proposed model’s $P_{d}$ reaches approximately 95%, whereas ParallelCNN and DeepSense remain at about the 92% level. This shows that the proposed model not only has higher overall accuracy but also provides the most efficient and stable trade-off between false alarms and detection rates, which is critical in practical detection systems.

Both experimental results show the superiority of the proposed IPFSCNN model. The performance gap between the proposed model and the existing models was particularly pronounced in low SNR environments. This is attributed to the dual-stream architecture of the IPFSCNN, which fuses dynamic features from the time domain (I/Q) with static features from the frequency domain (FFT) in a complementary manner. This approach enables more robust and accurate feature extraction in the presence of noise compared to models that rely on a single data representation.

### 4.2. Ablation Study for Architectural Validation

An ablation study was conducted to validate the design of the proposed IPFSCNN architecture by systematically removing or altering its key components and observing the impact on performance.

#### 4.2.1. Analysis of Component Contribution

**Figure 4:**
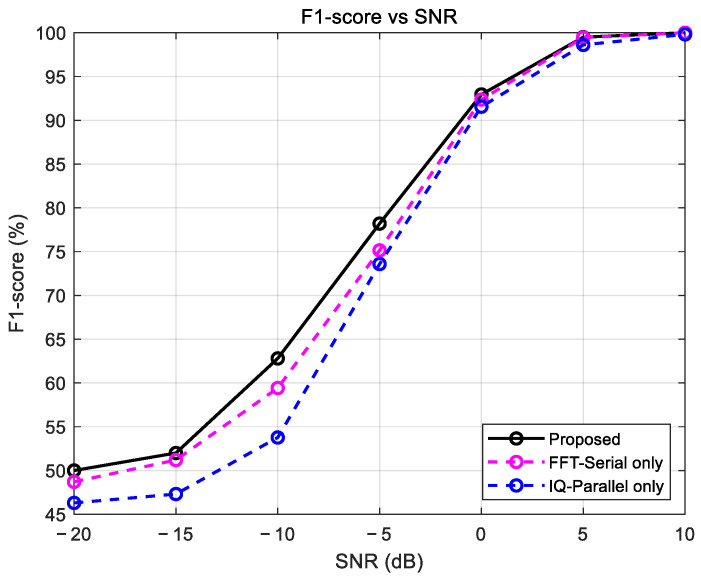
Performance contribution of each branch in the IPFSCNN model.

Figure 4 presents the results of an ablation study comparing the performance of the complete IPFSCNN model (“Proposed”) against versions that use only one of its branches (“IQ-Parallel only” or “FFT-Serial only”). The “FFT-Serial only” model, which uses the FFT-Serial branch, generally outperformed the “IQ-Parallel only” model, which uses the IQ-Parallel branch. This indicates that the explicit frequency-domain information is a primary contributor to the model’s overall performance.

Critically, however, the complete proposed model outperformed the “FFT-Serial only” model across all SNR conditions. This demonstrates that the IQ-Parallel branch provides complementary information from the time domain that the FFT branch cannot capture alone, resulting in a positive synergistic effect. This result validates the superiority of the hybrid, dual-stream design, as the combined architecture is more powerful than its individual components.

#### 4.2.2. Validation of Asymmetric Input Assignment

**Figure 5:**
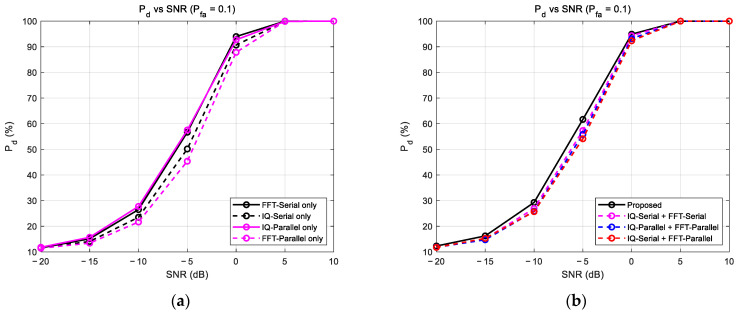
Detection performance according to architecture and data representation combination: (**a**) Individual performance based on the combination of architecture and data representation. (**b**) Overall model performance across various combinations.

To validate the proposed IPFSCNN’s architectural design, we conducted the ablation study shown in Figure 5. Figure 5a analyzes the individual performance of each branch (Serial and Parallel) paired with each data representation (I/Q and FFT). Figure 5b then compares the final performance of models using different fusion combinations of these branches.

Figure 5a shows which data representation each branch architecture is optimized for. The Serial Branch achieved superior detection performance when combined with FFT data (FFT-Serial only) compared to when it was combined with I/Q data (IQ-Serial only). For instance, at an SNR of −5 dB, the detection performance of ‘FFT-Serial only’ (black solid line) is approximately 7% higher than that of ‘IQ-Serial only’ (black dashed line). FFT data is refined information that explicitly shows the energy distribution across frequency bands, whereas I/Q data is raw data with a complex waveform over time. When I/Q data is fed into the Serial Branch, the model must first undertake the much more difficult learning task of extracting frequency information from the I/Q waveform. This process is inefficient, which is why the performance was superior when the more direct information from FFT data was input to the same architecture.

In contrast, the Parallel Branch achieved higher performance when combined with I/Q data (IQ-Parallel) than when combined with FFT data (FFT-Parallel). For instance, at an SNR of −5 dB, the detection performance of ‘IQ-Parallel only’ (magenta solid line) is approximately 10% higher than that of ‘FFT-Parallel only’ (magenta dashed line). To input FFT data into the Parallel architecture, the 128-point I/Q data must be split into two 64-point segments, and then a 64-point FFT is performed on each. This results in a halving of the frequency resolution compared to performing a full 128-point FFT on the entire signal. A lower spectral resolution degrades the ability to distinguish adjacent signals or capture fine signal features, which ultimately leads to a decrease in the model’s final performance. Therefore, the Parallel Branch’s performance was superior when combined with I/Q data.

The IPFSCNN is designed with an asymmetric architecture, where time-domain data (IQ) is routed to the Parallel branch and frequency-domain data (FFT) is routed to the Serial branch. To validate the effectiveness of this design, the performance of the proposed model was compared against models with various other combinations of branches and input data, with the results shown in Figure 5b. Our proposed IPFSCNN, which uses the asymmetric design (IQ-Parallel + FFT-Serial), outperformed all other fusion combinations. For instance, at an SNR of −5 dB, its detection performance (black solid line) was approximately 3–5% higher than these other combinations. This comparison demonstrates that merely fusing two data streams is insufficient. The key factor is how the data is fused; the best performance is achieved by combining feature extractors that are each optimized for their specific data type. This result proves that the high performance of IPFSCNN is not simply an artifact of using two branches, but a direct result of its intentional, asymmetric architecture, which applies the optimal processing method to each data’s unique characteristics.

### 4.3. Model Complexity

In addition to accuracy and robustness, we analyzed the complexity of each model to evaluate its feasibility for practical hardware implementation. The results are summarized in Table 2 was assessed based on two metrics: the number of learnable parameters, which relates to the model’s size and memory footprint, and the number of multiply-accumulate (MAC) operations, which relates to the computational workload during inference.

As shown in Table 2, our Proposed IPFSCNN model is significantly more efficient than the DeepSense baseline. It uses approximately 15% fewer parameters (33.6 K vs. 39.5 K), indicating a more efficient memory footprint. This efficiency is even more pronounced in computational load: IPFSCNN requires only 304 K MACs, achieving superior performance with roughly one-third of the computations needed by DeepSense (933 K).

Conversely, the ParallelCNN model is the most lightweight (20.8 K parameters, 102 K MACs). Our IPFSCNN is inherently more complex because it is a more sophisticated, dual-stream fusion model. However, as demonstrated in our ablation studies, the lightweight, I/Q-only architecture of ParallelCNN has performance limitations that cannot be overcome simply by increasing its size (e.g., adding more layers). IPFSCNN overcomes this architectural limitation by incorporating the FFT-Serial branch.

Therefore, the moderate increase in complexity over ParallelCNN is an intentional design trade-off to achieve the significantly higher accuracy that the simpler model cannot. Ultimately, IPFSCNN offers the most effective balance between high performance and computational efficiency, supporting its suitability for real-time applications where both are crucial.

## 5. Conclusions

In this paper, we proposed the IPFSCNN, a novel dual-stream architecture designed to enhance the performance of deep learning-based wideband spectrum sensing by synergistically fusing information from both the time domain (I/Q) and the frequency domain (FFT). The proposed model features a parallel architecture with two specialized branches: a Parallel Branch optimized for I/Q data processing and a Serial Branch optimized for FFT data processing, with their respective features effectively fused for a final classification.

Through various experiments on an LTE-M dataset, we demonstrated the superiority of the proposed model. The experimental results showed that the IPFSCNN outperformed the baseline models, DeepSense and ParallelCNN, across all SNR conditions. The performance gap was particularly significant in low-SNR environments. Furthermore, a detailed ablation study validated our design, confirming that each branch is specialized for its designated data representation and that their combination creates a synergistic effect that enhances the final performance. The complexity analysis results show that the proposed model is more efficient than the baseline models when considering both performance and complexity.

In conclusion, this study shows that the synergistic fusion of IQ-FFT data representations is an effective strategy for improving spectrum sensing performance. The proposed IPFSCNN architecture provides an accurate and efficient solution that contributes to the advancement of real-time, high-precision wideband spectrum sensing technologies. Future work could involve evaluating its generalization capabilities across a wider variety of communication signal environments.

## Figures and Tables

**Table 1:** Hyperparameters of the IPFSCNN.

Layers	Filter	Stride	Output Shape
I1/I2	-	-	(64, 2)
C1/C3 (LeakyRelu)	8 × 3	1	(62, 8)
M1/M3	2	2	(31, 8)
C2/C4 (LeakyRelu)	32 × 5	2	(14, 32)
M2/M4	2	2	(7, 32)
C5/C6 (LeakyRelu)	8 × 5	1	(120, 8)
M5	2	2	(60, 8)
C7/C8 (LeakyRelu)	16 × 11	1	(40, 16)
M6	2	2	(20, 16)
F1 (LeakyRelu)	224	-	64
F2 (LeakyRelu)	432	-	32
F3 (Sigmoid)	96	-	16

**Table 2:** Comparison of model complexity.

Layers	Proposed	DeepSense	ParallelCNN
Parameter	33,632	39,472	20,848
MACs (K)	304	933	102

## Data Availability

This work used the simulated LTE-M uplink dataset available on GitHub (version 1.0, https://github.com/wineslab/deepsense-spectrum-sensing-datasets (accessed on 10 October 2025)).
